# The m^6^A-ncRNAs axis in diabetes complications: novel mechanism and therapeutic potential

**DOI:** 10.3389/fendo.2024.1426380

**Published:** 2024-06-24

**Authors:** Siming Yu, Chunsheng Li, Xinxin Lu, Zehui Han, Yue Li, Xingxing Yuan, Dandan Guo

**Affiliations:** ^1^ Department of Nephrology II, First Affiliated Hospital of Heilongjiang University of Chinese Medicine, Harbin, China; ^2^ School of Graduate Studies, Heilongjiang University of Chinese Medicine, Harbin, China; ^3^ Department of Gastroenterology, Heilongjiang Academy of Traditional Chinese Medicine, Harbin, China; ^4^ Department of Cardiology, Second Affiliated Hospital of Heilongjiang University of Chinese Medicine, Harbin, China

**Keywords:** N^6^-methyladenosine, non-coding RNA, diabetic nephropathy, diabetic retinopathy, diabetic cardiomyopathy, diabetic angiopathy

## Abstract

Diabetes, a multifaceted metabolic disorder, poses a significant global health burden with its increasing prevalence and associated complications, such as diabetic nephropathy, diabetic retinopathy, diabetic cardiomyopathy, and diabetic angiopathy. Recent studies have highlighted the intricate interplay between N^6^-methyladenosine (m^6^A) and non-coding RNAs (ncRNAs) in key pathways implicated in these diabetes complications, like cell apoptosis, oxidative stress, and inflammation. Thus, understanding the mechanistic insights into how m^6^A dysregulation impacts the expression and function of ncRNAs opens new avenues for therapeutic interventions targeting the m^6^A-ncRNAs axis in diabetes complications. This review explores the regulatory roles of m^6^A modifications and ncRNAs, and stresses the role of the m^6^A-ncRNA axis in diabetes complications, providing a therapeutic potential for these diseases.

## 1 Introduction

Diabetes mellitus, a metabolic disorder characterized by chronic hyperglycemia, represents a significant global health burden due to various long-term complications including diabetic nephropathy (DN), diabetic retinopathy (DR), diabetic cardiomyopathy (DC), and diabetic angiopathy (DA) (1, 2). The pathophysiology of these complications is complex and multifaceted, involving biochemical, molecular, and cellular changes, among which post-transcriptional modifications of non-coding RNAs (ncRNAs) have emerged as crucial areas of interest (3). One of abundant and biologically significant modifications in RNA is N^6^-methyladenosine (m^6^A), affecting multiple aspects of RNA metabolism, such as stability, splicing, and translation (4). The dynamic and reversible modification of RNA by m^6^A is mediated by a set of proteins known as methyltransferases, demethylases, and binding proteins, which recognize and bind to m^6^A sites (5). The m^6^A modification landscape interacts significantly with ncRNAs, a diverse class of RNAs including microRNAs (miRNAs), long non-coding RNAs (lncRNAs), and circular RNAs (circRNAs), playing critical roles in regulating gene expression at the transcriptional and post-transcriptional levels (6, 7).

Emerging evidence underscores a pivotal role of the m^6^A-ncRNAs axis in regulating the pathological processes underlying diabetes complications. For instance, m^6^A modification influences the maturation, stability, and function of miRNAs, thereby participating in influencing cell survival in hyperglycemic state (8). Similarly, it also affects the structure and function of lncRNAs and circRNAs, which are involved in crucial regulatory pathways impacting inflammation, fibrosis, and endothelial dysfunction, which are key processes in the progression of diabetes complications (9, 10). The interplay between m^6^A modifications and ncRNAs opens new vistas for understanding the molecular mechanisms underpinning diabetes complications. However, the precise mechanisms by which m^6^A modifications affect ncRNA function in the context of diabetes are not fully elucidated, and the therapeutic potential of modulating this axis is still largely unexplored. Hence, this review concisely summarizes the regulatory mechanisms of m^6^A modifications and ncRNAs, and emphasizes current knowledge and recent discoveries concerning the role of the m^6^A-ncRNA axis in diabetes complications, aiming to provide a detailed understanding of this axis as a critical component in the pathogenesis of diabetes complications and its potential for therapeutic intervention.

## 2 The regulation of RNA m^6^A modification

The m^6^A modification is defined as methylation of the N6 position of adenosine and occurs in the common sequence DRACH (D = G/A/U, R = G/A, and H = A/C/U) (11, 12), which usually locates in the 3′-untranslated regions (UTRs), coding sequences, around stop codon regions, and 5′-UTRs (13, 14). This process orchestrates intricate regulatory networks on RNA, covering the reversible and dynamic interplay among methylases (writers), demethylases (erasers) and m6A-binding proteins (readers) (15).

The writers are at the forefront of m^6^A regulation and are responsible for the targeted deposition of m^6^A marks on RNA transcripts (16, 17). The core writer complex, comprised of methyltransferase-like 3 (METTL3) and METTL14, ensures the precise installation of m^6^A marks, underscoring the significance of this dynamic complex (16). Beyond the core writers, accessory proteins play pivotal roles in modulating m^6^A modification. Wilms tumor 1-associated protein (WTAP) has been identified as a key cofactor, facilitating the interaction between METTL3 and METTL14 and influencing their substrate specificity (18). Moreover, Vir-like m^6^A methyltransferase-associated (VIRMA, also known as KIAA1429), RNA‐binding motif protein 15 (RBM15) and its paralog RBM15B have been recognized as additional components of the m^6^A writer complex, expanding the regulatory landscape (19). Currently, novel methyltransferases, such as METTL4, METTL5, METTL16, and zinc finger CCCH domain‐containing protein 13 (ZC3H13), has been identified as modifiers to affect RNA metabolism in diabetes and its complications (20–22), showcasing their adaptability to diverse RNA substrates.

Equally pivotal in the m^6^A regulatory network are the erasers that specifically remove m^6^A marks. Two prominent erasers, fat mass and obesity-associated protein (FTO) and alkB homolog 5 (ALKBH5), serve as demethylases to catalyze RNA demethylation (23, 24). FTO, initially identified in the context of obesity-related processes, mediates the demethylation of m6A sites, resulting in the alteration of RNA structure and function (23, 25). ALKBH5, a member of the alkB family, specifically obliterates m^6^A marks in the coding region and 3’UTR of single-stranded RNA, and directly eliminate methyl groups from m^6^A-methylated adenosine (26). These erasers contribute to the dynamic turnover of m^6^A modifications, influencing various cellular processes, including mRNA stability and translation efficiency, contributing to glucose metabolic disorders in multiple organs and tissues, such as the liver, kidney, and blood vessels (27, 28).

Completing the triumvirate are the readers that recognize and interpret the m^6^A marks, transducing them into functional outcomes. Increasing m^6^A reader proteins have been discovered in mammals, including YTH domain‐containing family proteins (YTHDF1–3), YTH domain‐containing proteins (YTHDC1–2), insulin-like growth factor 2 mRNA-binding proteins (IGF2BP1–3), and heterogeneous nuclear ribonucleoproteins (HNRNPs) (29). YTHDF1 has been implicated in promoting the translation efficiency of m^6^A-modified mRNAs (30). YTHDF2, conversely, facilitates the degradation of m^6^A-modified transcripts through interactions with the RNA decay machinery, influencing mRNA stability (31). YTHDF3 cooperates with YTHDF1 and YTHDF2 to fine-tune the functions of m^6^A modification on gene regulation (32). Besides, YTHDC1, a nuclear m^6^A reader, mediates RNA splicing in the nuclear speckle and transports m^6^A methylated RNA from the nucleus to induce protein expression (33). YTHDC2 increases the translation efficiency of the substrate by targeting the conserved m^6^A motif (34). As another class of m^6^A readers, IGF2BPs are required for the stability of m^6^A-modified transcripts and enhance their translation, participating in activation of oncogenic pathways and resistance to targeted therapies (35). The HNRNP family of readers incorporates HNRNPC, HNRNPG and HNRNPA2B1, which bind to m^6^A sites of RNAs with high affinity. Both HNRNPC and HNRNPG affect the localization and splicing of RNAs in the nucleus (36), while HNRNPA2B1 regulates m^6^A modification via facilitating the processing of miRNA primers and miRNA precursors (37).

Conclusively, the dynamic interplay among m^6^A writers, erasers, and readers regulates fundamental cellular processes, including RNA processing, stability, and translation. In diabetes and its complications, the dysregulation of m^6^A modifiers is instrumental in determining the cellular RNA methylation landscape, ultimately affecting gene expression and biological processes, such as pancreatic β-cell dysfunction, insulin resistance, and abnormal lipid metabolism (5, 22, 38). As vital gene regulators, ncRNAs have also been implicated in the occurrence and progression of metabolic diseases. The intricate interplay between m^6^A modifiers and ncRNAs in the cellular milieu highlights the complexity of m^6^A modification in these diseases. Thus, targeting m^6^A modifiers holds substantial promise for the development of innovative therapeutics, provided that ongoing and future studies continue elucidating the mechanisms of these regulators in diabetes and its complications.

## 3 Regulatory mechanisms of ncRNAs

NcRNAs mainly contains miRNAs, lncRNAs, and circRNAs, playing a crucial role in the occurrence and development of diabetes complications (3, 39). NcRNAs are involved in the expression regulation of target genes via a variety of molecular mechanisms.

MiRNAs, a class of small endogenous and single-stranded ncRNAs with a length of 20–24 nucleotides, participate in regulating gene expression and affecting the development of various diseases (40). The biosynthesis of miRNAs is mediated by primary miRNA transcripts (pri-miRNAs) that are transcribed by RNA polymerase II, and pri-miRNAs are subsequently converted into precursor mRNAs (pre-mRNAs) by the Drosha–DGCR8 complex in the nucleus. Pre-miRNAs are next delivered into the cytoplasm and are further catalyzed to generate a nucleotide duplex by the RNase III enzyme Dicer, ultimately one strand of the duplex is processed by the RNA-induced silencing complex to produce a mature miRNA (41). By matching with the complementary sequences at the 3′-UTR of mRNAs of target genes, miRNAs suppress mRNAs translation or trigger their degradation, and thus regulating the post-transcriptional process of gene expression (42). In diabetes complications, the aberrant expression of miRNAs affects various biological process including cell proliferation, apoptosis, inflammation, angiogenesis, and tissue fibrosis, thereby acting as novel biomarkers and potential therapeutic targets (43, 44).

LncRNAs, a kind of transcripts of with lengths over 200 nucleotides and are transcribed by RNA polymerases, with no or limited protein-coding potential (45). Each lncRNA has distinct structures and functions, and its dynamic secondary and tertiary structures are required for their interchangeably interaction with DNA, RNAs, and proteins (45). LncRNAs have been implicated in gene expression regulation at various levels, ranging from transcription to post-translation. At the transcriptional level, lncRNAs are confirmed to modulate gene expression through interacting with chromatin-modifying enzymes and transcription factors (46, 47). Besides, at the post-transcriptional level, lncRNAs induce the inactivation or even degradation of mRNA by hindering the pre-mRNA chain splicing or directly binding to mRNA; meanwhile, they function as miRNA sponges, which is also referred to a competing endogenous RNAs (ceRNAs) mechanism, leading to the loss of regulatory function of miRNA on mRNA (48). Also, at the translation and post-translation level, lncRNAs act as protein scaffolds to mediate the assembly of translational components and form a specific spatial structure to activate or inhibit functions of proteins (49). Moreover, lncRNAs interact with signaling molecules and further master the activation of downstream signal transduction pathways (50). Abnormally expressed lncRNAs are observed in patients with various diabetes complications, which are considered to be potential diagnostic and prognostic biomarkers (51). In preclinical settings, dysregulation of lncRNA expression in animal models of diabetes complications is extensively engaged in multiple pathological processes, such as inflammation, oxidative stress, fibrosis, and microvascular dysfunction (52). Clarifying the regulatory mechanisms of lncRNAs in the pathogenesis of diabetes complications may provide new therapeutic avenues for this disease.

CircRNAs are a family of single-stranded covalently RNA molecules with the length of 100nt ~ over 4 kb, in which the closed loop is formed by the link between 5’ and 3’ terminal nucleotide sequences during splicing (53). With the help of the ligated 5′ and 3′ ends, circRNAs are able to avoid degradation mediated by exoribonuclease and are steadier compared to linear transcripts such as miRNAs and lncRNAs (54), indicating a potential for diagnostic or prognostic markers of various diseases. Like these linear RNAs, circRNAs are also derived from pre-mRNAs and are transcribed by RNA polymerase II (RNA Pol II), which are further regulated by the cis-regulatory elements and trans-acting factors, suggesting a competition with linear RNAs during their biogenesis (55). In fact, circRNAs have been verified to modulate gene expression via various mechanisms. First, they serve as miRNA sponges directly targeting miRNAs by competing with mRNA, thus modulating gene expression. Besides, they function as transcription regulators of target genes through interacting with RNA Pol II by a mechanism of cis-regulation. Finally, they serve as protein scaffolds to block protein function and inactivate related signaling pathways (56, 57). Increasing studies have proved the aberrant expression of circRNAs in patients with diabetes complications, implying its feasibility as a diagnostic or prognostic biomarker for these diseases (58, 59). Experimentally, circRNAs are susceptible to high glucose (HG)-induced tissue injury, and resultant dysregulation of their expression levels and functions contribute to the pathological process under diabetic conditions (60, 61). Therefore, circRNAs exhibit potential roles as novel biomarkers and treatment tools in diabetes complications.

**Figure 1:**
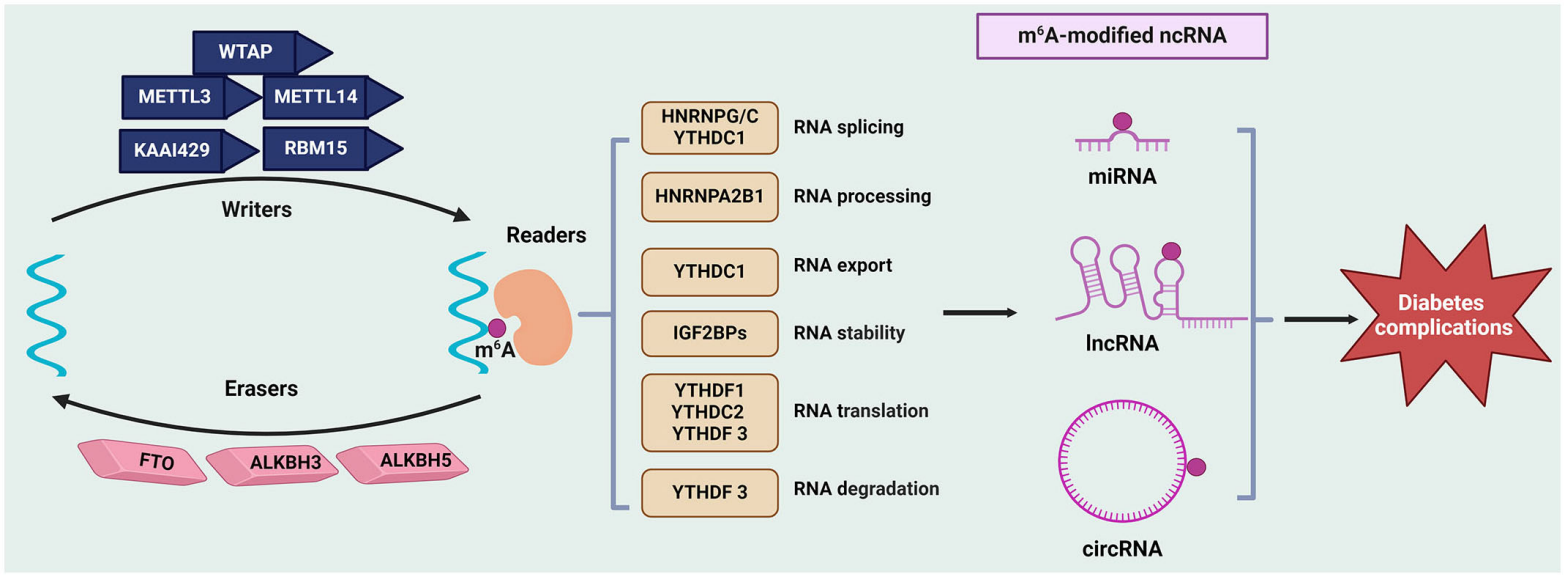
Regulation of ncRNAs modifications by m^6^A in diabetes complications. The m^6^A modification is mediated by writers (such as METTLs, WTAP, KAAI429, and RBM15), erasers (FTO, ALKBH5, and ALKBH3), and readers (like YTHDFs, YTHDCs, IGF2BPs and HNRNPs). This modification affects the metabolism ncRNAs (include miRNAs, lncRNAs, and circRNAs), such as their stability, splicing, and translation, thus regulating gene expression and further influencing the initiation and progression of diabetes complications. ALKBH, AlkB Homolog; circRNAs, circular RNAs; FTO, Fat Mass and Obesity-Associated Protein; HNRNPs, Heterogeneous nuclear ribonucleoproteins; lncRNAs, long non-coding RNAs; METTL, methyltransferase‐like; miRNAs, microRNAs; ncRNAs, non-coding RNAs; m^6^A, N6-methyladenosine; RBM15, RNA‐binding motif protein 15; KAAI429, Vir‐like m6A methyltransferase associated; WTAP, Wilms’ tumor 1-associating protein; YTHDF, YTH domain‐containing family.

To sum up, miRNAs directly target mRNAs to regulate the gene translational process. Both lncRNAs and circRNAs function as ceRNAs to sponge miRNAs, thus affecting the miRNAs-mediated gene regulation. They also directly interact with proteins, influencing protein functions and their downstream signaling pathways. The expression of ncRNAs is dysregulated in diabetes complications where ncRNAs are engaged in various biological processes, such as insulin secretion, vascular dysfunction, thermal hyperalgesia, tissue fibrosis, and cellular autophagy. As concluded above, m^6^A modification also participates in the onset and progression of diabetes complications through affecting these pathological processes. As a main target of m^6^A methylation, the modification of ncRNAs may play a fundamental role in their regulation, protein interactome and subsequent downstream effector functions (7, 62), exerting a crucial role in the initiation and progression of diabetes complications (**Figure 1**). In this context, further investigating roles of m^6^A-regulated ncRNA might provide potential diagnostic or prognostic markers for diabetes complications, as well as promising therapeutic targets to tackle this disease.

## 4 The m^6^A-modified ncRNAs in the pathogenesis of diabetes complications

### 4.1 Diabetic nephropathy

DN stands as a prominent microvascular complication of diabetes mellitus, leading to end-stage renal disease and heightened mortality rates among affected individuals (63). Podocytes, a kind of epithelial cells that located outside the glomerular capillaries, act as a crucial component of the glomerular filtration barrier, thus podocyte injury is regarded as the core event in the occurrence and progression of DN (64). Recent research endeavors have shed light on the intricate molecular mechanisms underlying DN pathogenesis, with a particular focus on the involvement of ncRNAs and their m^6^A modifications. For example, METTL14 is found to promote renal tubular epithelial cell apoptosis and endoplasmic reticulum stress during DN progression by regulating the m^6^A modification of lncRNA TUG1. This interaction facilitates the activation of MAPK/ERK signaling, further exacerbating DN pathogenesis (65). Additionally, the upregulation of lncRNA ENST00000436340, mediated by FTO-induced m^6^A modification, promotes podocyte injury by facilitating the association of polypyrimidine tract binding protein 1 (PTBP1) with the Rab GTPases RAB3B mRNA, leading to cytoskeleton rearrangement and inhibition of glucose transporter 4 (GLUT4) translocation (66). Further analysis of macrophage M1-related lncRNAs in the process of m6A modification identifies several lncRNAs including LINC00342, LINC00667, and LNC00963 are regulated by FTO, RBM15, and WTAP, which is required for the immune regulation of macrophage M1 during DN progression (67). Also, m^6^A modification of circRNAs has emerged as a critical regulatory mechanism in DN progression. It is reported that METTL3 reduces the expression of circRNA 0000953 by upregulating its methylation level through YTHDF2, which inhibits podocyte autophagy via the miR665–3p/ATG4b (autophagy-related gene 4b) axis, thereby exacerbating podocyte injury and albuminuria in DN mice (68). Similarly, circRNA UBXN7 promotes macrophage infiltration and renal fibrosis via interactions with IGF2BP2-dependent specificity protein 1 (SP1) mRNA stability, suggesting its potential as a prognostic indicator and therapeutic target for DN management (9). These findings underscore the circRNA-mediated regulatory networks orchestrated by m^6^A modifications in DN pathogenesis.

Collectively, m^6^A-modified ncRNAs have emerged as key players in modulating various aspects of DN progression, including podocyte injury, macrophage infiltration, renal fibrosis, and inflammatory responses. Targeting these m^6^A-related modifiers and their associated regulatory networks may hold the key to mitigating the progression of DN and improving patient outcomes. Future research endeavors aimed at unraveling the complexities of m^6^A-modified ncRNAs in DN pathogenesis are warranted to facilitate the development of novel therapeutic strategies for this debilitating condition.

### 4.2 Diabetic retinopathy

DR represents a major microvascular complication of diabetes mellitus and a leading cause of vision loss. Recent advances in molecular biology have unveiled the critical roles of m^6^A modifications and ncRNAs in the pathophysiology of DR (69), which have been identified as key regulators in various biological processes, including angiogenesis, inflammation, and cell death (70). Research has elucidated multiple mechanisms through which m^6^A modifications of ncRNAs influence DR progression. For example, a study identified that METTL3 overexpression attenuates HG-induced pyroptosis in retinal pigment epithelial cells by regulating the miR-25–3p/PTEN/AKT signaling cascade. This process enhances cell viability and reduces oxidative stress responses, crucial for preventing DR exacerbation (8). Another aspect of METTL3 roles in DR is demonstrated through the induction of mature miR-4654, which facilitates HG-mediated apoptosis and oxidative stress in lens epithelial cells via decreasing the expression of antioxidant superoxide dismutase 2 (SOD2) (71). In addition, METTL3 mediates m^6^A modifications enhancing the stability of lncRNA SNHG7, which inhibits the endothelial-mesenchymal transition via downregulating the RNA-binding protein KHSRP and the transcriptional coactivator MKL1, thus mitigating DR progression (72). Another lncRNA, NEAT1 is upregulated by WTAP in DR, and further aggravates cell apoptosis and inflammation through activating the nod-like receptor pyrin domain 3 (NLRP3) inflammasome (73). Additional findings have highlighted the role of circRNA FAT1 in DR. This circRNA is confirmed to enhance autophagy and further decrease pyroptosis in retinal pigment epithelial cells under diabetic conditions through its interaction with YTHDF2 (74). Hence, the diversity of these mechanisms underscores the complexity of regulatory roles of m^6^A on ncRNAs and their broad therapeutic potential in DR.

In sum, the detailed mechanisms by which m^6^A modification controls ncRNA function and stability provide a deeper understanding of DR at a molecular level, suggesting that targeting specific m^6^A writers, erasers, or readers could offer new strategies for treatment. Future research should focus on elucidating more comprehensive pathways involving m^6^A-modified ncRNAs, validating their roles *in vivo*, and exploring their potential in clinical applications.

### 4.3 Diabetic cardiomyopathy

DC represents a severe and often irreversible cardiac dysfunction linked to diabetes, primarily characterized by fibrosis, apoptosis, and metabolic disturbances (75). It is reported that the expression of lncRNA Airn is decreased in HG-treated cardiac fibroblasts, which contributes to myocardial fibrosis and cardiac dysfunction. The anti-fibrotic effect of lncRNA Airn is mechanistically linked to its interaction with IGF2BP2, protecting p53 mRNA from degradation in an METTL3-dependent manner, thereby leading to cell cycle arrest in fibroblasts and reduced cardiac fibrosis (76). Similarly, the role of METTL14 is explored in its capacity to suppress pyroptosis and ameliorate DC progression by downregulating the lncRNA TINCR, which in turn upregulates the expression of NLRP3 by increasing its mRNA stability (77). Conversely, circRNA CDR1as is verified to promote cardiomyocyte apoptosis in DC. Mechanistically, ALKBH5 cooperates with YTHDF2 to enhance the expression of transcription factor FOXO3, which is responsible for the upregulation of circRNA CDR1as, thus inhibiting the ubiquitination of mammalian sterile 20-like kinase 1 (MST1) and further activating of the Hippo signaling pathway (78). These findings provide insights into the regulatory mechanisms of m^6^A modification on ncRNAs in DC.

In brief, these studies underscore the significant regulatory roles of m^6^A-modified ncRNAs in the pathophysiology of DC. The intricate molecular interactions point to a promising frontier in cardiac research where ncRNAs and epigenetic modifications intersect to offer novel insights and therapeutic strategies in the fight against DC. Future research should aim to expand the repertoire of ncRNAs and m^6^A modifications associated with DC, exploring their interactions and the downstream pathways they regulate. This could pave the way for the development of multi-target drugs that modulate several pathways simultaneously, offering a more holistic approach to managing or potentially curing DC.

### 4.4 Diabetic angiopathy

DA, a critical complication of diabetes, manifests predominantly as vascular dysfunction leading to morbidity and mortality among diabetic patients (79). Emerging research elucidates the significant role of m^6^A modifications in ncRNAs, which pivotally influence vascular cell behavior and disease progression in diabetic conditions. For instance, one such study focused on human umbilical vein endothelial cells, treated with HG and TNF-α to simulate diabetic endothelial dysfunction, revealed extensive changes in the m^6^A landscape of lncRNAs, which is involved in the regulation of metabolic processes and hypoxia-inducible factor 1 (HIF-1) signaling pathway, thus determining endothelial cell survival and function (80). Concurrently, another study investigated the role of circRNA YTHDC2 in vascular smooth muscle cells, a vital player in vascular integrity and remodeling (10). In this preclinical setting, circRNA YTHDC2 is overexpressed and its depletion suppresses cell proliferation and migration, thereby alleviating vascular remodeling and atherosclerosis. Furthermore, the stability of circRNA YTHDC2 is regulated via YTHDC2-mediated m^6^A modification; subsequently, circRNA YTHDC2 is verified to inhibit the expression of ten-eleven translocation 2 (TET2), a regulator of DNA demethylation, which prompts a dedifferentiated phenotype in vascular smooth muscle cells, contributing to diabetic vascular complications (10).

**Figure 2:**
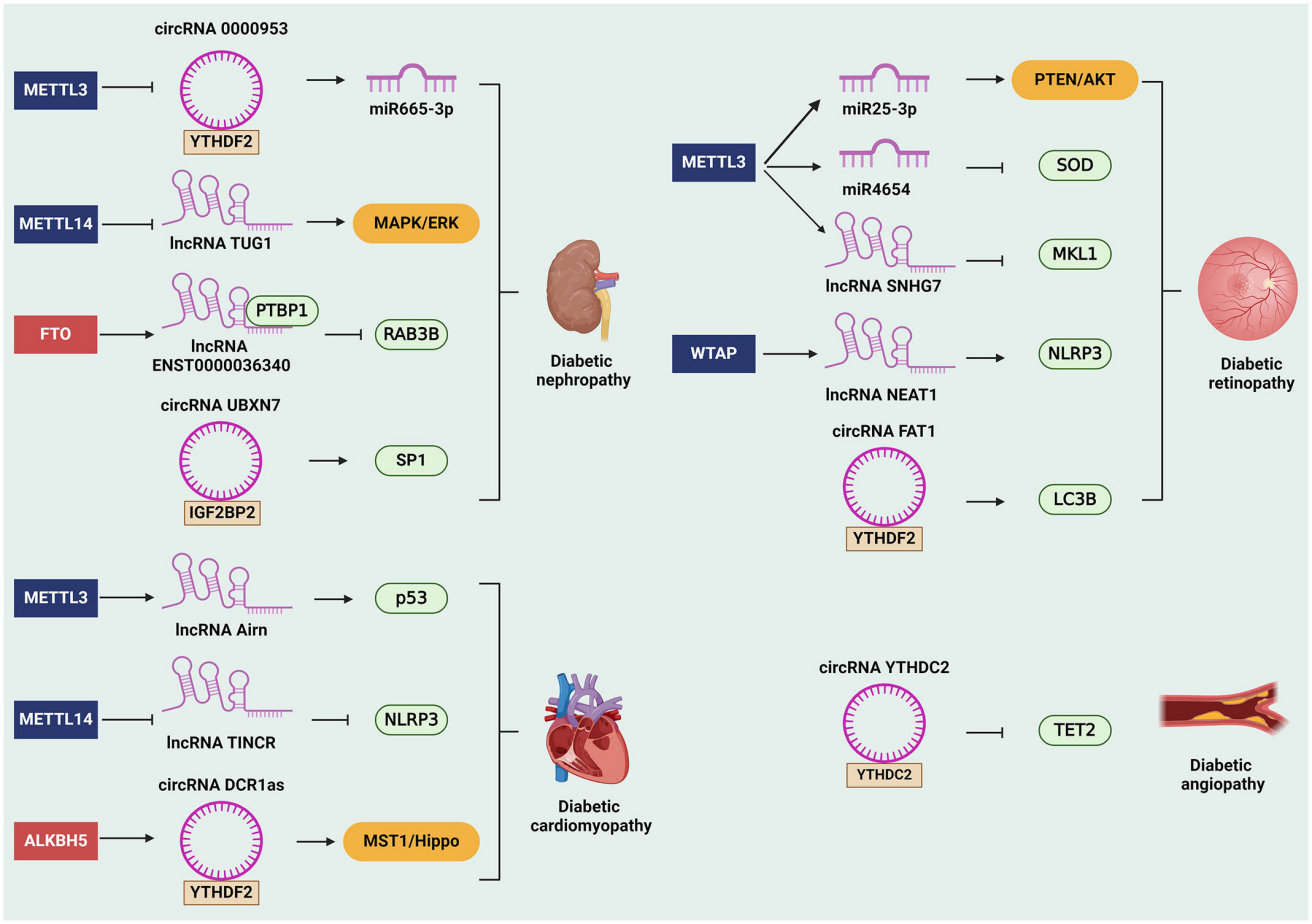
The role of m^6^A-modified ncRNAs in diabetes complications. The m^6^A-modified ncRNAs are involved in the occurrence and development in diabetes complications. For instance, m^6^A- modified ncRNAs can affect diabetic nephropathy by regulating downstream genes and signaling pathways, such as miR665–3p, RAB3B, SP1, and MAPK/ERK signaling pathway. Besides, m^6^A-related ncRNAs promote diabetic retinopathy by regulating SOD, MKL1, NLRP3, LC3B, and PTEN/AKT signaling pathway. Moreover, m6A-related ncRNAs facilitate the progression of diabetic cardiomyopathy via modulating the expression of p53 and NLRP3. Likewise, m6A-related ncRNAs can also affect the development of diabetic angiopathy through inhibiting the expression of TET2. ALKBH5, alkB homolog 5; circRNAs, circular RNAs; FTO, fat mass and obesity-associated protein; IGF2BP2, insulin-like growth factor 2 mRNA-binding protein 2; lncRNAs, long non-coding RNAs; m^6^A, N6-methyladenosine; METTL, methyltransferase-like; miRNAs, microRNAs; MST1, mammalian sterile 20-like kinase 1; ncRNAs, non-coding RNAs; NLRP3, nod-like receptor pyrin domain 3; PTBP1, polypyrimidine tract binding protein 1; SP1, specificity protein 1; SOD2, superoxide dismutase 2; TET2, ten-eleven translocation 2; WTAP, Wilms tumor 1-associated protein; YTHDF2, YTH domain‐containing family protein 2; YTHDC2, YTH domain‐containing protein 2.

In conclusion, m^6^A modifications affect ncRNA function and resultant vascular cell behavior in diabetes. The intricate network of m^6^A-modified ncRNAs modulating key signaling pathways and cellular behaviors presents a promising therapeutic frontier (**Figure 2**). By targeting specific ncRNAs and related m^6^A modifications, it might be possible to mitigate or even reverse the pathological vascular remodeling characteristic of DA. Of interest, in type 2 diabetes patients, it has been demonstrated that HG enhances FTO mRNA expression and further decreases the m^6^A level, which is responsible for the upregulation of methyltransferases, suggesting that glucose participates in the dynamic regulation of m^6^A (81). However, whether the dynamic of HG-mediated m^6^A modification affects downstream ncRNAs in diabetes complications should be taken into account in future investigation.

## 5 Conclusions and future perspectives

This review synthesizes the current knowledge regarding the regulation of m^6^A modification on ncRNAs in diabetes and its complications, as well as emphasizes the roles of m^6^A-modified ncRNAs on these diseases, shedding light on novel mechanisms and therapeutic avenues. The dysregulation of m^6^A modifications in ncRNAs, including miRNAs, lncRNAs, and circRNAs, has emerged as a key driver in the pathophysiology of diabetic nephropathy, retinopathy, cardiomyopathy, and angiopathy. By modulating the expression and function of critical genes involved in insulin signaling, oxidative stress, inflammation, and cellular metabolism, aberrant m^6^A-ncRNAs axis contributes to the progression of diabetes complications. Therefore, further understanding of the molecular intricacies underlying m^6^A-mediated ncRNA regulation provides a foundation for the development of targeted interventions aimed at restoring homeostasis and ameliorating diabetes complications. It should be noted that therapeutic potentials of targeting the m^6^A-ncRNAs axis in diabetes complications lies in its ability to modulate gene expression at the post-transcriptional level with precision and specificity. Strategies targeting m^6^A writers (e.g., METTL3, METTL14), erasers (e.g., FTO, ALKBH5), and readers (e.g., YTHDF1–3, YTHDC1–2) offer promising avenues for therapeutic intervention. Small molecule inhibitors, RNA-based therapeutics, and CRISPR-based approaches hold immense potential in restoring the dysregulated m^6^A modification patterns in diabetic tissues, thereby attenuating disease progression and improving clinical outcomes.

However, several challenges and opportunities for future research warrant consideration. Firstly, the tissue-specific regulation of m^6^A modifications in ncRNAs underscores the importance of understanding context-dependent effects on diabetes complications. Comprehensive profiling of m^6^A landscapes across different tissues and cell types will enhance our understanding of spatial and temporal dynamics in diabetic pathogenesis. Secondly, elucidating the crosstalk between m^6^A-modified ncRNAs and other epigenetic mechanisms, such as DNA methylation and histone modifications, may uncover synergistic or antagonistic interactions driving diabetes complications. Hence, harnessing the regulatory potential of m^6^A modifications in ncRNAs might reshape the landscape of diabetes care, advancing towards precision medicine tailored to the individual needs of diabetic patients.

## Author contributions

SY: Writing – original draft, Conceptualization. CL: Writing – review & editing. XL: Writing – review & editing. ZH: Writing – review & editing. YL: Writing – review & editing. XY: Writing – original draft, Conceptualization. DG: Writing – original draft, Conceptualization.
